# Exploring an Unknown Corner of a Well-Known Topic: HIIE Influence on Renal Health and Filtration in Healthy Individuals Free of Cardiometabolic Diseases

**DOI:** 10.3390/sports11110210

**Published:** 2023-10-30

**Authors:** Jeffrey S. Forsse, Kathleen A. Richardson, Ricardo Torres, Catherine Lowry, James Kyle Taylor, Cassidy L. Beeson, Jacob Ward, Anurag Dhillon, Brock Niceler, Ahmed Ismaeel, Panagiotis Koutakis

**Affiliations:** 1 Integrated Laboratory of Exercise, Nutrition, and Renal Vascular Research, Department of Health, Human Performance, and Recreation, Baylor University, Waco, TX 76706, USA; katie_adair1@baylor.edu (K.A.R.); ricardo_torres1@baylor.edu (R.T.); catherine.lowry@colostate.edu (C.L.); clbeeson5@gmail.com (C.L.B.); jward42@siumed.edu (J.W.); anu.dhillon10@gmail.com (A.D.); brock.niceler@wacofamilymedicine.org (B.N.); 2 Department of Health and Exercise Science, Colorado State University, Fort Collins, CO 80011, USA; 3 Medical & Clinical Laboratory Sciences, Auburn University—Montgomery, Montgomery, AL 36124, USA; jtaylor@aum.edu; 4 Southern Illinois University Medical School, Lindegren Hall, 600 Agriculture Dr #132, Carbondale, IL 62901, USA; 5 Health Science Center, San Antonio Joe R and Teresa Lozano Long School of Medicine, The University of Texas, 7703 Floyd Curl Drive, San Antonio, TX 78229, USA; 6 Waco Family Medicine, Waco, TX 76707, USA; 7 Department of Physiology, University of Kentucky, 780 Rose Street, MS508, Lexington, KY 40536, USA; ahmed.ismaeel@uky.edu; 8 Clinical Muscle Biology Lab, Baylor University, Waco, TX 76706, USA; panagiotis_koutakis@baylor.edu

**Keywords:** renal health and filtration, contemporary biomarkers, high-intensity interval exercise

## Abstract

Aerobic exercise, specifically high-intensity interval exercise (HIIE), and its effects on renal health and filtration (RHF) are not well understood. Several studies support incorporating contemporary biomarkers serum cystatin C (CyC) and urine epidermal growth factor (uEGF) to combat the volatility of serum creatinine (sCr). Using these biomarkers, we examined the acute influences HIIE has on RHF to determine if there is a ceiling effect in healthy populations. The purpose was to determine the influence of an acute bout of HIIE on RHF. Thirty-six participants (*n* = 22 males; *n* = 14 females; age 37.6 ± 12.4 years.; BF% 19.2 ± 7.1%; VO_2_max 41.8 + 7.4 mL/kg/min) completed 30 min of HIIE on a treadmill (80% and 40% of VO_2_reserve in 3:2 min ratio). Blood and urine samples were obtained under standardized conditions before, 1 h, and 24 h post-exercise. CyC, sCR, uEGF, urine creatinine (uCr), uCr/uEGF ratio, and multiple estimates of glomerular filtration rate (eGFR) Modification of Diet in Renal Disease (MDRD) and CKD-EPI equations were used. The analysis employed paired sample t-tests and repeated measures ANOVAs. CyC, uEGF, uCr, and uCr/uEGF ratio concentrations were not altered between timepoints. sCr increased 1 h post-exercise (*p* > 0.002) but not at 24 h post-exercise. eGFR decreased in the MDRD and CKD-EPI equations at 1 h (*p* > 0.012) with no changes at 24 h post-exercise. CyC and sCr/CyC demonstrated no significant changes. CyC and uEGF are not altered by acute HIIE. The results demonstrate a potential ceiling effect in contemporary and traditional biomarkers of RHF, indicating improvements in RHF may be isolated to populations with reduced kidney function.

## 1. Introduction

The mechanisms of renal decline observed in people with chronic kidney disease (CKD) still elude academics and clinicians [1,2,3]. This process is primarily due to the lack of signs and symptoms in early disease progression, and early signs of CKD are non-specific and difficult to diagnose [4,5]. Decline in renal function has long been linked to the aging process and considered to be a by-product of aging based on creatinine clearance [6,7]. However, current research suggests that in contrast to conventional clinical knowledge and practice, renal decline may not progress as rapidly with age [3]. Instead, it appears interconnected to underlying cardiometabolic diseases (CMDs) (e.g., hypertension (HTN), obesity, dyslipidemia, diabetes, sedentary lifestyle, smoking, and family history) that contribute to the current rate of kidney decline (8–16%), which is a growing prevalence in adults worldwide [5,8,9,10]. High morbidity and mortality rates make CKD a global issue [11] and are further exacerbated by the lack of knowledge on the early progression of renal decline [5]. Thus, the lack of early diagnosis of CMD causes a decline in renal tubular health (function) and filtration (RHF) in at-risk populations [3,12,13].

Serum creatinine (sCr) has been the method of choice for assessing renal filtration for years due to its relative non-invasive procedure and cost-effectiveness [14]. However, circulating creatinine concentrations vary significantly with age, sex, muscle mass, medications, diet, and select disease states [15,16]. Due to the high volatility of creatinine, more contemporary biomarkers cystatin C (CyC) and urine epidermal growth factor (uEGF) have been growing in popularity among researchers and clinicians [17,18,19]. CyC appears to be more accurate than creatinine as it is not secreted into renal tubules [20]. CyC is produced by all cell types in the body, providing a more accurate assessment of indirect measures of renal filtration [18,21]. Additionally, contemporary biomarker uEGF is produced in the ascending limb of the Loop of Henle and distal convoluted tubule, permitting a more direct measurement of renal health [22,23]. An increased production of uEGF initiates intracellular pathways that cause upregulation in renal cell survival, growth, and replication [24].

Researchers’ understanding of exercise’s influence on modulating biomarkers of RHF has continued to improve [4,25]. One area that has gained more interest is the sympathetic nervous system (SNS) and its effects on the kidneys. The shunting of blood away from the kidneys during periods of high SNS activity, such as during high-intensity interval exercise (HIIE) has become an area of increasing interest [26]. Previous studies utilized an acute bout of moderate-intensity continuous exercise (MICE) on biomarkers of RHF in healthy individuals and an acute HIIE and continuous moderate-intensity exercise bout in moderate stages of CKD [1,4,25]. There is a greater improvement in RHF observed in individuals with CKD when compared to healthy individuals after aerobic exercise, which remains unchanged. However, the effects of HIIE and its influence on RHF in healthy individuals free of CMD remain unexplored when utilizing new methods and biomarkers for assessment. Presently, the literature on this subject is limited to select populations, and the underlying mechanisms of renal decline are not fully understood. Therefore, employing sCr in conjunction with CyC and uEGF in measuring RHF in apparently healthy individuals free of CMD will allow us to differentiate the effects of HIIE on RHF more clearly.

This study aimed to determine the influence of an acute bout of HIIE on traditional and novel biomarkers of RHF in healthy individuals. We hypothesize an acute bout of HIIE will have minimal effect on RHF in apparently healthy people free of CMD based on the near-optimal function of the kidneys in healthy individuals.

## 2. Materials and Methods

### 2.1. Subject Enrollment and Demographics

**Figure 1:**
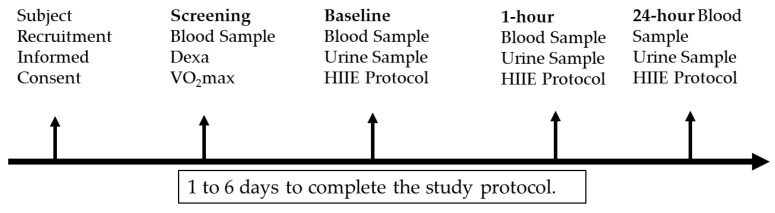
Research timeline.

Subjects recruited were apparently healthy, free from cardiometabolic risk factors (confirmed), physically active, non-smokers, not pregnant, with normal body fat percentages, between the ages of 20 and 60, with no diagnosed diseases, and attended regular physical assessments by a physician. A total of 45 individuals were screened for the study, with 36 individuals (males = 22 and females = 14) participating and completing the study (See Figure 1). Nine participants did not meet entry into the study due to failure to meet inclusion criteria (e.g., disease diagnosis, time commitment, age, and cardiorespiratory fitness). Participant demographics are provided in Table 1. Physically active is defined as greater than 150 min of exercise per week [27]. All participants were able to perform vigorous exercise.

### 2.2. Ethical Approval

University Institutional Review Board (IRB) for research with human subjects (project #) grant approval was obtained before the start of data collection. The research study and protocol comply with the ethical guidelines outlined in the 1975 Declaration of Helsinki. All participants were given written material regarding the research study, which was verbally explained to each participant. The informed consent was authorized and returned by participants before admittance into the study.

### 2.3. Baseline Physiological Assessment

A health history questionnaire (e.g., cardiovascular, metabolic, respiratory, physical activity, family history, etc.) and physiological assessments (e.g., heart rate, blood pressure, CBC, CMP, lipid panel, Dexa) measured overall health status and quantified renal health and filtration for all participants. Participants were instructed to abstain from exercise 48 h before the health assessment. All vitamins, supplements, and non-steroidal anti-inflammatories were not consumed during the study protocol period. Baseline and exercise heart rates were recorded using a Polar H7 heart rate monitor (Polar, Bethpage, NY, USA). Blood pressure was obtained manually (American Diagnostic Corporation, Hauppauge, NY, USA) by experienced technicians. A baseline blood sample was obtained from the most prominent vein in the antecubital space to determine cardiometabolic health (e.g., lipid profile, complete metabolic panel, and complete blood count).

All subjects completed a maximal oxygen consumption (VO_2max_) exercise treadmill test to determine cardiorespiratory fitness classification. Respiratory gases (VO_2_ and VCO_2_) were continuously assessed using indirect spirometry via a respiratory gas analysis system (TrueOne 2400™, ParvoMedics^®^, Sandy, UT, USA). Cardiopulmonary metrics (e.g., heart rate, respiratory gases, and VO_2max_) were monitored throughout the test. The VO_2max_ test consisted of one minute of standing data to obtain baseline respiratory values. Followed by a ramp protocol treadmill test, speed was determined using the participant’s predetermined 5 K race pace (participants indicated how fast they believed they could run a 5 K). Speed was increased 1 mph every three minutes for the first three stages. The incline grade was at 0% for the first three stages, and the grade was raised by 2% increments every minute thereafter, with speed remaining constant after stage three until the end-of-test criteria were reached [1,28]. Obtaining a true VO_2max_ required a respiratory exchange ratio (RER) of >1.1, a plateau of VO_2_, rate of perceived exertion ≥ 17, and/or achieving 85% or higher of age-predicted max heart rate [29]. The test was terminated if the exercise physiologist witnessed signs or symptoms that justified termination the exercise test or upon the subject’s request. All participants returned to the lab another day to complete the exercise intervention protocol for a minimum of 2 days following the screening session.

### 2.4. HIIE Exercise Protocol

**Figure 2:**
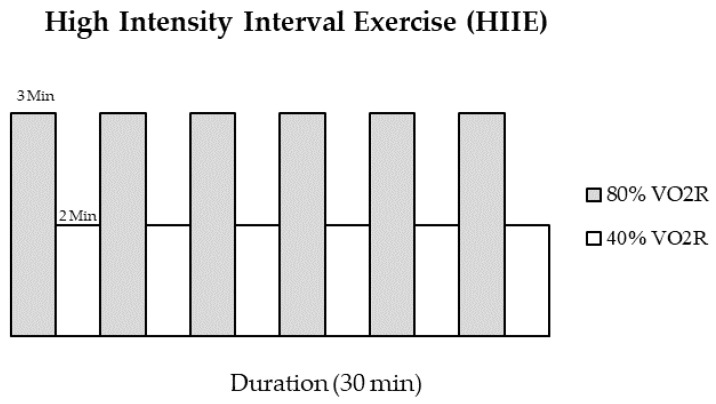
Exercise protocol.

Subjects arrived at the research lab after a minimum of an eight-hour fast limited to water ingestion only to maintain proper hydration before and during (16 oz) the exercise protocol. Participants were instructed not to consume coffee, caffeine, or alcohol on the days of the research study. Participants wore standard fitness attire (shorts, t-shirt, and running shoes). Resting heart rate and blood pressure was obtained before exercise in a seated position. The experimental exercise condition consisted of 30 min of HIIE. During HIIE, all participants completed six stages of high and low levels of intensity. Each stage included 3-min intervals of submaximal yet vigorous exercise at 80% of peak VO_2_Reserve (VO_2_R) and 2-min recovery intervals of walking at 40% VO_2_R (Figure 2). Heart rate and respiratory gases were continuously monitored throughout the exercise session.

### 2.5. Specimen Collection

Blood samples were acquired prior to the exercise session and again at 1 h and 24 h post-exercise. Blood samples, equal to about 1.1 tbsp (17 mL), were obtained by venipuncture in the left arm’s most prominent vein site in the antecubital space. Serum samples were allowed to clot for 30 min at room temperature. In contrast, plasma EDTA samples were stored on ice for 30 min, which was followed by centrifugation at 3330 RPM for 10 min for both serum and plasma. Finally, serum and plasma samples were separated and aliquoted into 2 mL microplastic storage tubes and stored at −80 °C until all data were collected and analyzed.

Urine samples were collected in a sterilized container at baseline, 1 h, and 24 h post-exercise. Participants were sent to the restroom with a specimen cup and asked to void their bladder into the sample collection container. Upon receiving the collected urine sample, the samples were placed on ice for 30 min and centrifugated for five minutes at a lower speed of 1000 RPMs to prevent protein damage. Samples were separated and aliquoted into 1.5 mL microplastic storage tubes and stored at −80 °C until all data were collected and ready to be analyzed.

To account for changes in hydration between timepoints, hematocrit was assessed to determine shifts in plasma volume. Subjects were recommended to adhere to their standard diet practices and fluid consumption, caffeine and alcoholic beverages were not allowed during the 24 h period.

### 2.6. Biochemical Analysis

Biomarkers sCr and CyC were analyzed using the Piccolo Xpress blood chemistry analyzer Comprehensive Metabolica Panel (Abaxis, Inc., Union City, CA, USA) and a commercial ELISA kit (R&D Systems, Minneapolis, MN, USA and Arbor Assays, Ann Arbor, MI, USA), respectively. Intra-assay precision for the ELISA kit was determined to have a 3.1% coefficient of variation (CV). eGFR (mL/min/1.73 m^2^) was determined using equations validated by the National Kidney Foundation [3,30]. uEGF concentrations were determined using a commercial ELISA kit (R&D Systems, Minneapolis, MN, USA) with an intra-assay precision of 2.5% CV. Urine creatinine (uCr) concentrations were determined by a colorimetric detection kit (Enzo Life Sciences Inc., Farmingdale, NY, USA). Serum, plasma, and urine sample were allowed to thaw to room temperature before analysis. All samples, controls, and standards were assayed in duplicates. The ELISA kit plate well optical density was determined using an ELx808 absorbance microplate reader set to 450 nm (Biotek, Winooski, VT, USA). The uCr/uEGF ratio was log_2_ transformed to normalize the results. Estimates of renal filtration were calculated using the Modification of Diet in Renal Disease (MDRD) (GFR in mL/min per 1.73 m^2^ = 175 × SerumCr^−1.154^ × age^−0.203^ × 1.212 (if patient is black) × 0.742 (if female)) and CKD epidemiology (GFR = 141 × min(Scr/κ, 1)α × max(Scr/κ, 1) − 1.209 × 0.993Age × 1.018 [if female] _ 1.159 [if black], where Scr is serum creatinine, κ is 0.7 for females and 0.9 for males, α is −0.329 for females and −0.411 for males) equations using biomarkers sCr and CyC (See Table 2) [31].

### 2.7. Statistics

Statistical significance for all tests was set at a priori at the ≤0.05 level. The power calculation was based on a power (1-β error probability of 0.90) with an effect size of 0.25, resulting in the need for 36 individuals to be sufficiently powered. Significant differences between variables (e.g., markers of renal health and filtration and age) were determined by repeated-measures ANOVA. T-tests were used to determine significant differences and distribution. A Tukey’s post hoc analysis was used to determine all possible pairwise comparisons since the young vs. older groups were unequal. A Pearson’s coefficient was used to calculate the relationship between markers of renal health and filtration with age. Data were analyzed with SAS software version 9.4 (SAS, Cary, NC, USA).

## 3. Results

### Renal Biomarker Outcomes

Relative to pre-exercise measurements, sCr significantly increased 1 h post-exercise (*p* = 0.0024) while the comparison between pre- and 24 h post-exercise sCr returned to baseline values, exhibiting no significant changes (see Table 2 and Table 3). uCr significantly increased from 1 to 24 h post-exercise. The concentrations of CyC and uEGF did not show significant changes across any timepoints. When comparing the pre-exercise and 24 h post-exercise time periods, none of the biomarker concentrations were significantly changed. sCr was the only biomarker to demonstrate a significant difference based on the effects of time. No biomarkers demonstrated significant differences based on the effects of age or the age * time interaction. There were no significant shifts in plasma volume between timepoints. All data were normally distributed.

## 4. Discussion

To our knowledge, this is the first study to examine the effects of HIIE on contemporary and traditional biomarkers of RHF in healthy individuals free of CMD. The primary findings of our study show that an acute bout of HIIE did not alter contemporary biomarkers of RHF CyC and uEGF across all time intervals. sCr significantly increased at 1 h post-exercise when compared with pre-exercise concentrations. However, we did not observe any significant changes in sCr between pre- and 24 h post-exercise concentrations. Collectively, these observations support CyC and uEGF as more accurate measures when determining exercises’ acute influence on RHF in healthy individuals free of CMD.

Conventional biomarker sCr transiently increased 1 h post-exercise (Figure 2). This finding is similar to the current literature on aerobic exercise and sCr [32,33,34]. However, the mode of exercise is different; thus, a direct comparison between previous studies cannot be fully elucidated. Nevertheless, the likelihood of significant differences between higher-intensity modes of aerobic exercise on sCr concentrations is minimal. Our lab recently published a study looking at the effects of an acute bout of MICE on biomarkers of RHF [4]. The present study’s results coincide well with our previous study’s results. The ephemeral elevations in sCr concentration post-exercise were within the expected outcomes. These transient elevations are believed to be mainly due to the body’s transition from the SNS shunting of blood away from the kidneys, which was followed by a reactive hyperemic response to replenish the kidney’s blood supply for filtration [1], resulting in a transient increase in free circulating sCr in the blood by increases in skeletal muscle catabolism. Therefore, the increase in sCr concentrations appears to be directly related to skeletal muscle degradation and not changes in renal filtration. This outcome and rationale are further supported by changes in uCr, which significantly decreased at 1 h post-exercise and then returned above baseline at 24 h (see Table 2 and Table 3).

The utilization of CyC as a more reliable biomarker of renal filtration continues to gain support among researchers and clinicians [18,21,35]. More specifically, using CyC to assess changes in renal filtration during and following exercise continues to gain momentum amongst researchers [4,36,37]. In our present study, we did not observe any elevations of CyC, which is different from previous studies performed by Forsse et al. [4] and Bonger et al. [38] who observed acute elevations in CyC post-exercise. Forsse et al. [4] utilized an acute bout of MICE (for 20 min) at 60% of VO_2max_R in healthy males and females and observed a 14% increase from baseline. In contrast, Bongers et al. [38] implemented a 150 min acute cycle session set at 80% of maximal heart rate in healthy male adults. The elevation seen in the previous studies is possibly due to the previous studies acquiring blood measurements at 30 min post-exercise. We hypothesize by the 1 h post-exercise timepoint that the CyC has already been filtered by the kidneys. Had we measured CyC 30 min post-exercise in the current study, we expect it would have been elevated. Thus, no elevations were observed in CyC, and both studies and the current study support the growing science that CyC is a less volatile measurement of RHF than sCr.

Contemporary biomarker uEGF did not significantly increase at any timepoint post-exercise (See Table 2). These results are consistent with Forsse et al. [4] who observed no significant increases in uEGF after an acute bout of moderate-intensity aerobic exercise in healthy individuals free from CMD. However, the study outcomes also differ from Konradsen et al. [39], who implemented a two-hour cross-country race in 25 healthy, trained individuals and observed an increase in uEGF concentrations immediately post-exercise. The main differences between all three studies that preclude a direct comparison are the duration and intensity of the exercise sessions. Forsse et al. [4] utilized an exercise session of 20 min of sustained moderate-intensity exercise inside on a treadmill, while Konradsen et al. [39] used a two-hour race outside, with participants stating that they had finished the race at maximal effort. Although the results of our study did not present any significant changes in uEGF, the differences in means at 1 h were 9.7% higher when compared to baseline and were 6.4% higher at 24 h when compared to baseline. However, no significant changes were observed due to the large standard deviations. Furthermore, our study also utilized the uEGF/uCr and Log_2_ (uEGF/uCr) equations to better evaluate renal health compared to renal filtration, which resulted in no significant changes in uEGF/uCr or Log_2_ (uEGF/uCr) between the pre-exercise and 1 h post-exercise time period (see Table 3), which is consistent across all three studies that recruited healthy individuals. However, when sampling CKD populations with reduced RHF, the results are different. Forsse et al. [25] implemented an acute bout of aerobic exercise in individuals diagnosed with moderate stages (G3a-b) of CKD. There was an observed significant increase (*p* = 0.049) in the uEGF/uCr ratio when implementing acute bouts of HIIE set at an intensity of 90% and 20% of VO_2_R (3 min and 2 min) for 30 min. However, an additional acute bout of continuous moderate-intensity exercise (65% VO_2_R) for 30 min elicited no significant changes in the uEGF/uCr ratio. Additionally, based on the raw concentrations of baseline uEGF of healthy individuals compared to CKD individuals, healthy individuals had 4× the baseline concentrations of uEGF as CKD individuals, further indicating diminished renal health in CKD individuals and suggesting the potential reason for significant differences in uEGF/uCR ratio responses to exercise. When considering the methodologies and results of all the studies, it appears that acute improvements in RHF are associated with intensity and duration of exercise as well as renal health status.

Multiple estimates of renal filtration provide a more in-depth assessment of RHF outcomes. Based on the sample population, all standard recommended eGFR equations were utilized (see Table 2 and Table 3). Calculated values for sCr MDRD, sCr CKD-EPI, and eGFR sCr/CyC significantly decreased over the pre-exercise to 1 h post-exercise time period, which coincides with changes observed in sCr concentrations post-exercise. The results are consistent with previous studies that observed decreases in eGFR when using sCr as the indirect biomarker to assess renal filtration in healthy populations [4,32,33,34,40,41]. Consequently, the aforementioned research studies noted that the greatest changes in sCr and eGFR are mainly due to the intensity and length of the exercise session that activates a more significant amount of skeletal muscle catabolism [32,36,40]. The average decline in eGFR was ameliorated during the hours post-exercise, returning to baseline within 24 h. Therefore, changes in renal filtration via eGFR do not appear to be directly related to damage to the glomeruli following HIIE in healthy populations. Based on eGFR calculated solely from CyC, there was no change in renal filtration in the hours post-exercise (*p* = 0.5741 and *p* = 0.9382), further supporting the theory of a minimal to no amount of damage to the glomeruli in individual with healthy renal function. Additionally, the results further support the clinical significance of incorporating CyC as a standard biomarker of overall RHF that is resistant to high variability in healthy and diseased individuals at risk for CKD and renal decline.

Limitations of our study include (1) using indirect methods like CyC and sCr to assess RHF, (2) participants reporting their health information correctly and accurately, (3) relying on participants to abstain from exercise for 24 h before the exercise session, (4) trusting participants to remain hydrated as described in the protocol, (5) an unequal number of male and female participants, and (6) comparing the exact results to different modes of exercise. Future studies should focus on the primary avoidance of renal decline by exploring the prevention of CMD and developing recommendations for proper renal health. More research should also be conducted to describe the effects of exercise in increasing RHF in people with renal decline and CKD.

## 5. Conclusions

This study demonstrated that in healthy individuals without CMD, renal health does not appear to be acutely altered by an acute bout of HIIE. Contemporary biomarkers, CyC and uEGF, do not significantly change with exercise of differing time intervals, indicating a more stable assessment of RHF with exercise. Hence, there may exist a potential ceiling effect in RHF when healthy individuals participate in aerobic exercise of differing intensities and modalities compared to CKD individuals who have reduced RHF. Our research team considers this study as an enhancing piece to the existing collection of knowledge on contemporary and traditional biomarkers of RHF. We believe implementing CyC, uEGF, and uEGF/uCr ratios in clinical settings will allow researchers and clinicians to accurately measure acute and chronic changes in RHF in different patient populations.

## Figures and Tables

**Table 1:** Participant demographics.

Variable	Mean	SD
**Age (yrs)**	37.6	12.4
**Height (cm)**	172.8	9.4
**Weight (kg)**	73.3	15.3
**Body Fat Percentage (BF%) (%)**	19.2	7.1
**Systolic Blood Pressure (SBP) (mmHg)**	119.9	6.9
**Diastolic Blood Pressure (DBP) (mmHg)**	77.1	5.9
**Resting Heart Rate (HR) (beats per min)**	60.0	11.5
**Exercise HR (beats per min)**	177.5	18.2
**VO_2max_ (mL/kg/min^−1^)**	41.8	7.4
**Glucose (mg/dL)**	95.0	6.5
**Total Cholesterol (Chol) (mg/dL)**	178.1	32.4
**HDL (mg/dL)**	58.5	13.7
**LDL (mg/dL)**	102.6	26.9
**Albumin (g/dL)**	4.7	0.2

Data are presented as mean + SD.

**Table 2:** Biomarker timepoint concentrations.

Variables	Baseline	1-h	24-h
**sCr (mg/dL)**	0.93 ± 0.15	0.97 ± 0.17	0.92 ± 0.15
**sCr MDRD**	84.88 ± 16.09	81.13 ± 16.05	86.31 ± 17.35
**sCr CKD-EPI**	97.02 ± 30.83	92.94 ± 30.57	95.81 ± 26.76
**eGFR CyC**	115.17 ± 52.6	111.97 ± 51.49	114.81 ± 47.1
**eGFR sCr/CyC**	104.4 ± 28.03	100.3 ± 26	102.75 ± 26.9
**uCr (mg/dL)**	212.51 ± 103.24	187.78 ± 117.2	230.13 ± 124.67
**CyC (mg/dL)**	873.9 ± 415.34	896.81 ± 443.23	862.44 ± 423.59
**uEGF (pg/mL)**	31,874.94 ± 19,457.88	34,973.52 ± 50,846.85	33,904.31 ± 21,158
**uEGF/uCr (ng/mL)/(mg/dL)**	15.23 ± 7.52	19.9 ± 31.55	15.26 ± 7.04
**Log_2_ (uEGF/uCr)**	1.33 ± 0.46	1.4 ± 0.84	1.34 ± 0.43

Abbreviations: Serum creatinine (sCr), modification of diet in renal disease (MDRD), chronic kidney disease–epidemiology (CKD-EPI), estimated glomerular filtration rate (eGFR), cystatin C (CyC), urine creatinine (uCr), and urine epidermal growth factor (uEGF). All equations used for calculating eGFR are presented in the units of mL/min/1.73 m^2^.

**Table 3:** Biomarkers of renal health and filtration.

Variable	Pre- and 1 h	1 h & 24 h	Pre- & 24 h	Effect of Time	Effect of Age	Age * Time Interaction	ES 1 h	ES 24 h
**sCr**	0.0024 *	<0.0001 *	0.2385	0.0002 *	0.8022	0.6074	−0.24	0.15
**sCr MDRD**	0.0031 *	0.0033 *	0.4522	0.0062 *	0.0532	0.6028	0.23	−0.08
**sCr CKD-EPI**	0.0122 *	0.5559	0.8114	0.5919	0.6028	0.1775	0.13	0.04
**CyC (eGFR)**	0.5741	0.5874	0.9382	0.7892	0.4607	0.1323	0.06	0.007
**sCr/CyC (eGFR)**	0.0067 *	0.3768	0.5603	0.2421	0.0268 *	0.6225	0.15	0.06
**uCr**	0.2091	0.0248 *	0.3911	0.1007	0.7973	0.9950	0.22	−0.15
**CyC**	0.4513	0.2425	0.6381	0.4637	0.2478	0.6711	−0.05	0.02
**uEGF**	0.7584	0.8974	0.6351	0.9279	0.2921	0.5783	−0.08	−0.09
**uEGF/uCr**	0.3632	0.3747	0.9999	0.4310	0.2176	0.3547	−0.20	−0.04
**Log_2_ (uEGF/uCr)**	0.6088	0.6896	0.9324	0.8227	0.2481	0.3192	−0.10	−0.02

“*” indicates a statistically significant value (*p* < 0.05). Abbreviations: Serum creatinine (sCr), Modification of Diet in Renal Disease (MDRD), chronic kidney disease-epidemiology (CKD-EPI), estimated glomerular filtration rate (eGFR), cystatin C (CyC), effect size (ES), urine creatinine (uCr), and urine epidermal growth factor (uEGF). All equations used for calculating eGFR are presented in the units of mL/min/1.73 m^2^.

## Data Availability

Not applicable.
